# A Review of the Human Health Risks from Microbial Hazards in Recreational Beach Sand

**DOI:** 10.3390/ijerph22101537

**Published:** 2025-10-08

**Authors:** Nicola King, Margaret Leonard

**Affiliations:** New Zealand Institute of Public Health and Forensic Science, Christchurch Science Centre, 27 Creyke Road, Ilam, Christchurch 8041, New Zealand

**Keywords:** beach sand, faecal indicator, recreational water, bathing, public health

## Abstract

At many recreational beaches, the health of visitors is protected through water quality monitoring programmes. However, visitors may also be exposed to microbiological pathogens in sand via ingestion, inhalation and skin contact. Microbiological pathogens that can cause human illness may be naturally found in beach sands, or introduced with people, animals or water entering the beach. The World Health Organization has recommended that recreational water safety plans consider microbial pathogens in beach sand. This review shows that a range of faecal and non-faecal pathogens can be detected in beach sand, but difficulty in determining whether exposure occurred via the sand or water means that there is insufficient evidence to link their presence with adverse human health effects. Proactively integrating beach sand testing into recreational water safety programmes will generate data to assess the impact of risk management activities. The use of faecal indicator bacteria to indicate elevated risk from faeces should be a priority where there are potential sources of contamination. This should be complemented with sanitary surveys and analyses that elucidate faecal contamination sources. The inclusion of non-faecal pathogens into monitoring programmes needs further, locally relevant justification through evidence from epidemiological studies and human health risk assessment.

## 1. Introduction

In 2021, the World Health Organization (WHO) published updated guidelines for assessing and monitoring recreational water quality for risks to public health such as gastrointestinal illness (GI), respiratory illness, wound infection and skin complaints [1]. It also recommended that risk factors for microbial pathogens in beach sand be incorporated into a recreational water safety plan. The recommended indicator organisms to assess risk were intestinal *Enterococcus* spp., as an indicator of recent faecal contamination and therefore the potential presence of pathogenic microorganisms. The guideline value for intestinal enterococci was 60 colony forming units (CFU)/g (wet weight) of sand, estimated based on rates of sand ingestion by children with *pica* tendencies and the assumption that the enterococci:pathogen ratios are the same in sand and water. The WHO suggested that additional microbiological guideline values could be set based on local characteristics and an assessment of public health risk, underpinned by epidemiological studies and quantitative microbial risk assessment (QMRA). The WHO recognised the need for more research to establish exposure thresholds for other microorganisms, and that species of bacteria, viruses, parasites or fungi could all be considered.

The presence of faecal indicator bacteria (FIB) in the environment generally indicates recent faecal contamination, and therefore the potential presence of pathogenic microorganisms also excreted with faeces [2]. FIB are used to indicate a potential risk to human health from faecal contamination since routine monitoring for the presence of microbial pathogens is impractical due to the high analytical costs, less robust methodology than for FIB and the variable prevalence in the community. *Escherichia coli* and *Enterococcus* spp. are consistently present in high concentrations in the faeces of warm-blooded animals, although in differing quantities [3]. Guideline values for enterococci are used to indicate the suitability of marine water for recreational contact, although *E. coli* may be used for freshwaters [1,4,5]. Enterococci best indicate health risks where the faecal contamination is dominated by human sewage [6]. However, it cannot be assumed that the presence of these FIB in water reflects the microbial health risks from nearby beach sand.

Building on the WHO’s 2021 guidelines, Brandão and others [7] have provided advice for sand sampling and testing regimes that could be considered for routine monitoring or outbreak investigations. They also comment that it is increasingly easy to detect pathogens directly, unlocking practical options for monitoring the non-faecal associated microorganisms.

However, the microbiological analytes and guidance values need to be locally relevant. This review was initiated to support recreational water managers in the temperate Pacific island nation of Aotearoa New Zealand to consider how the microbiological risks from beach sand could be monitored within existing public health environmental monitoring programmes.

## 2. Scoping for Local Relevance

This review was guided by three locally relevant factors: Climate, the nature of beach recreation and the communicable disease profile.

Studies conducted in temperate climate zones were prioritised, this being the “temperate-without a dry season” (Cf) zone according to the Köppen–Geiger climate classifications [8]. Data from other climate zones were included in place of absent data or to impart important findings.

**Figure 1:**
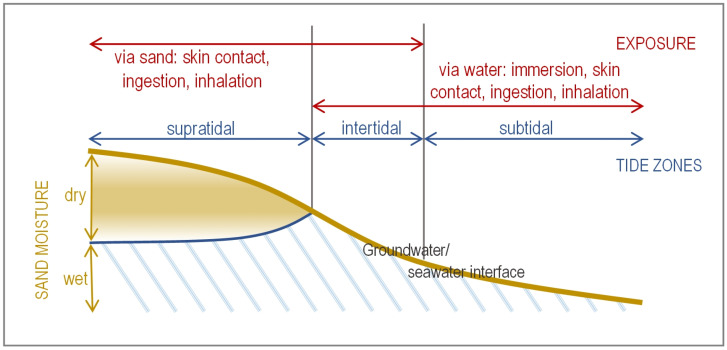
The three main zones of a coastal sandy beach (blue arrows and text) and the pathways for human exposure to microbial pathogens (red arrows and text).

Recreational beach sand was considered to be the sand in beach environments most likely to be visited by people for recreational purposes, where such visits often involve extended time spent in contact with the sand, e.g., lying on the beach, children playing with sand. Like Brandão et al. [7], this review focused on the intertidal (swash) and supratidal zones of coastal beaches, where human exposure to microbiological contaminants occurs via sand and water (depending on their activity) or dry sand, respectively (Figure 1). This review also considered beaches adjacent to non-tidal rivers and lakes where the main exposure is contact with dry sand (equivalent to the supratidal zone). While water in the subtidal zone can be shallow depending on the beach morphology, swimming is a common activity in this area so exposure to microbial pathogens is more likely to be through water contact (Figure 1).

The third lens applied to this review was the prevalence of relevant communicable diseases in the local population [9]. The scope was restricted to the causative microbiological pathogens of endemic communicable diseases.

## 3. Microorganisms in the Beach Environment

Sand can protect microorganisms against sunlight, trap organic matter and provide colonisable surfaces [10]. Microbial populations in the sand are natural inhabitants (autochthonous) mixed with temporary residents introduced from elsewhere (allochthonous) [11]. The microorganisms making up these communities vary widely, even across one location [12]. Introduced microorganisms might die quickly, might survive for days or months or, in the case of bacteria, might establish replicating populations and become naturalised.

Microbial transport in the beach environment is affected by the direction and energy of water and wind movements [11]. Microorganisms can be planktonic in water (including pore water between substrate grains) or attached to substrates where they can become part of protective biofilms [13]. Porous or fractured sand grains provide a suitable substrate for microbial attachment, as do environmentally ubiquitous microplastic and nanoplastic particles [14,15]. The region below the surface of the sand is saturated. Microorganisms within this space break down organic matter and undertake a wide range of biogeochemical reactions [16].

Faecal contamination of beach sand presents the highest risk of beach visitors being exposed to pathogenic microorganisms. However, there is currently limited epidemiological evidence connecting human infection or illness with sand exposure (Section 6). Point sources of faecal contamination include direct defaecation (e.g., by humans, companion animals, farmed animals or wildlife) and areas where faecal matter is concentrated, such as bird nesting areas, horse riding routes and toilets. Sewage and stormwater outflows from infrastructure are also point sources, although the impact of these on the microbial safety of the sand depends on the quality and dispersal of the discharge. For example, upgrading the stormwater infrastructure at a beach in Miami, USA, significantly improved sand and water quality, as measured by enterococci concentrations [15].

Non-point source discharges include other waterways, groundwater or stormwater. These can carry contaminants from land runoff, inland point source discharges and discharges to groundwater such as on-site wastewater systems or effluent irrigation. Urban centres close to recreational beaches can potentially increase non-point source contamination from human activities (e.g., failing sewer infrastructure, run-off from hard surfaces). It can be assumed that animals on farm or tourist operations adjacent to recreational beaches may also contribute non-point source contamination if faecal matter is washed into the beach area. Non-point sources may be more difficult to identify and control [17].

Finally, opportunistic windblown or waterborne pathogens may be naturally found in beach environments. Pathogenic microorganisms could also be introduced by people entering the beach environment, e.g., carried on their skin or introduced with shoes and vehicles.

## 4. Faecal Indicators in Beach Sands

As expanded below, FIB are useful indicators for the potential presence of pathogenic microorganisms in beach sand. FIB can be present at higher concentrations and survive better in sand compared to water, representing an ongoing contributor to FIB in the swim zone. Two main challenges for using FIB are their spatial variability (concentrations vary widely between and within beach zones) and the potential for false-positive results from naturalised populations. This spatial variability makes it difficult to correlate sand FIB concentrations with water FIB concentrations, environmental variables or public health outcomes, which is important for modelling and beach hygiene management. Methods to distinguish FIB from fresh faeces, aged faeces (i.e., faecal contamination in the past) and naturalised populations are needed. Complementary genomic methods, including those designed to identify sources of faecal contamination, offer solutions. This would provide clarity over health risks when environmental samples contain elevated numbers of *E. coli* but no obvious source of faecal contamination. Rapid, affordable methods to determine health risks from non-faecal microorganisms are also needed. Metagenomic methods may be suitable but the results need to be relevant for public health.

### 4.1. Faecal Indicator Bacteria

Studies of FIB in sand provide clues as to where enteric pathogens might also be found on sandy beaches. The general limitations to interpreting the relationship between FIB numbers in recreational water and human health risks also apply to sand. FIB do not confirm that enteric pathogens are present nor indicate the source of faecal matter, FIB may be poor indicators for pathogenic microorganisms that survive longer in environmental samples and are not indicators for non-faecal associated pathogens, and the strength of FIB as a marker for health risk diminishes in waters receiving low levels of diffuse, non-human or mixed faecal contamination [2,18,19,20]. A systematic review identified 10 studies that did not find a significant correlation between FIB concentrations in marine/brackish water and concentrations (or the presence/absence) of pathogenic microorganisms [18]. A further six studies reported a positive relationship between at least one indicator (most often enterococci) and one pathogen (most often adenoviruses, *Salmonella* spp., protozoan parasites or *Campylobacter* spp.). Correlations were stronger when FIB concentrations were high and faecal contamination was known to have occurred.

Naturalised (persistent) *E. coli* and enterococci have been identified in environmental matrices including soil, sediment and aquatic vegetation [2]. Devane et al. [2] view naturalised strains as two groups: Those that are defecated into the environment and able persist under favourable conditions by adapting to a non-host lifestyle (indicating past faecal contamination), and those that are truly environmental (separate lineages that lack the genes important for survival in the gut of animals). Others have described potentially self-sustaining *E. coli* populations in freshwater and coastal beaches, which were perhaps periodically refreshed by wildlife faeces or beach visitors [21,22]. The FIB populations might be distinguishable from naturalised populations using genomics but not by current standard FIB test methods [2]. However, work conducted since using *E. coli* isolates from beach sand, sewage and gull waste suggests that the genes important for long-term environmental sand survival might be widespread among *E. coli* strains [23].

The concentration of FIB in the water is not a reliable indicator of the concentration in sand on the adjacent, comparatively heterogenous beach. Some studies have reported significantly positive correlations between FIB concentrations in sand and water but with particular sample types and timescales [24,25,26]. Spatially, the concentration of FIB in sand can be highly variable. One study has investigated this variability at a microspatial level, finding that samples of dry sand taken every 10 cm along a 2 m transect could contain non-detectable levels of enterococci or concentrations as high as 5 × 10^2^ CFU/g [27]. Some of this variability is likely due to faecal deposits from wildlife, particularly birds, since enterococci concentrations could exceed 10^2^ CFU/g sand at the point of deposition [27].

Microcosm studies indicate that FIB could survive longer in sand compared with seawater [28]. Field studies show that the concentration of FIB in sand can be several orders of magnitude higher than the water [29,30,31], noting that comparisons between FIB concentrations in sand and water should be made with caution, considering the ratio of FIB:pathogen may differ and the two matrices require different sampling and testing methods. Environmental survival is improved when microorganisms, including FIB, attach to sediments in aquatic environments and become incorporated into biofilms [32,33,34,35,36], although this may affect their recovery during laboratory testing [7]. One study found a non-linear correlation between the sand concentrations of enterococci and extracellular polymeric substances (EPS), the main constituent of biofilms, but only in the supratidal zone [37].

#### 4.1.1. Environmental Factors Affecting FIB Survival

Reviews of FIB survival in sand [11], and in aquatic sediments [35,38] have explained the range of abiotic and biotic factors that combine to affect survival. Moist conditions, cooler temperatures and lower levels of solar irradiance favour microbial survival and may support bacterial growth, but this is not consistent between studies. Laboratory studies and field surveys have shown better survival and higher concentrations of natural populations of *E. coli* and enterococci in marine sands with relatively higher moisture [39,40]. However, laboratory and field studies have also shown that the concentration of enterococci (but not *E. coli*) in sand is either not significantly affected by moisture levels, or correlates negatively [37,41]. Laboratory studies suggest that high temperatures (>50 °C) will challenge FIB survival in sand [41,42]. Outdoors, sunlight introduces both heat and UV radiation into the sand with the latter damaging microorganisms, at least in the top few centimetres of the sand. During a beach study in Miami (USA) [43], the supratidal sand reached 40 °C on average (range 24–53 °C) but conditions remained suitable for enterococci survival, suggesting temperature exerts a weaker effect on FIB survival compared to sunlight-associated desiccation and UV.

From a three-month study of a Massachusetts beach (USA) [44], moisture and solar irradiance were the most informative variables for predicting enterococci concentrations in dry (supratidal) sand. Higher enterococci concentrations were associated with higher sand moisture (this ranged from <1% to 4%), increasing sand moisture (a wider tidal range, higher relative humidity) and lower solar irradiance (i.e., less sunshine). However, the researchers did not identify any environmental conditions that could predict when wet (intertidal) sand had elevated enterococci concentrations, although wind appeared to cause higher wave runup and recirculation of enterococci in the swash zone.

FIB can also be eaten by microfauna such as protozoa and nematodes, and face competition from other sand microflora [2,11]. In sediment microcosm experiments, FIB survived better in sterilised sand compared to non-sterilised sand [45,46]. Competition with autochthonous sand bacteria might be more important than predation [47], although other studies have found that predation dominates [38].

Nutrients from organic matter can be washed into the sand from the land or water, including large deposits of algae (seaweed, wrack). These encourage bacterial survival and growth by providing nutrients and protection from sunlight and desiccation. A study in New Zealand found high concentrations of enterococci in beach seaweed and a significant association between enterococci levels measured in the sand and in the seaweed [48]. A study in California (USA) found that the concentrations of enterococci and *E. coli* were higher in mixed macroalgae wrack and under-wrack sand sampled from the dry sand zone, compared to equivalent samples collected from the wet and surf zone areas [49]. Laboratory studies confirm that seaweed wrack supports FIB survival and growth in sand [48,49]. Beach grooming was found to reduce *E. coli* concentrations in the sand and improve water quality [50,51].

#### 4.1.2. ‘Hot Spots’ of Contamination

Hotspots of higher FIB concentrations can develop on the beach. One study found that enterococci from a single faecal deposit from a seagull could radially migrate in dry sand, contaminating an area of 3 m^2^ [27]. It has also been observed that *E. coli* persists on toilet wipes buried in sand, even when these degrade [52]. In the absence of visitors to a beach in Florida (USA), enterococci concentrations in the sand were highest in areas of the beach with stranded seaweed [43]. At times, enterococci concentrations exceeded the provisional guideline of 60 CFU/g sand [1]. When beach visitors returned, enterococci concentrations in other parts of the beach increased, particularly in the supratidal zone, and this was partly attributed to seaweed being spread by foot traffic. It was thought that enterococci were being washed out of the sand in the intertidal and subtidal zones.

As combinations of factors may cause FIB hot spots on a beach, it is not surprising that the findings of studies show considerable variation. A review of studies examining the fate of *E. coli* and enterococci in beach environments [11], showed that FIB densities could differ between beach zones (Figure 1) but not in a consistent pattern, i.e., some studies found higher FIB densities in wet foreshore sand compared to dry backshore sand, others found higher concentrations in lower moisture, supratidal sands. Other studies reaffirm these differences between beach zones [53,54]. This means that it is difficult to predict which beach zone will have higher FIB concentrations since every beach will be different. This is not unexpected considering the variability of beach environments and their catchments.

Despite the above, the intertidal zone might become a hot spot of contamination on low wave energy beaches, or during periods of low wave energy on surf beaches. Sand can serve as a vehicle for transferring pathogenic microorganisms to the water and can become contaminated by the water [55]. This mixing occurs through water movement (wave action, currents, interstitial pore water transport). Field experiments using synthetic microspheres as a proxy for bacteria indicated that high energy wave action in the swash zone rapidly moves non-attached (planktonic) bacteria deeper into the sand, and horizontally seaward [56]. In contrast, beaches with lower wave energy and longer slopes can allow bacteria to settle, aggregate and perhaps multiply [57]. A temporal study of a freshwater beach in Canada recorded lower concentrations of *E. coli* in foreshore sand samples during periods of higher wave heights [58]. Erosion of the sand into the water was the primary *E. coli* transport process at this fine sand beach. Interstitial pore water transport appeared to be more important on coarser sand beaches. Further evidence comes from surveys that have found higher concentrations of FIB in the wet sand of sheltered beaches compared with open beaches [10,22], although point and non-point discharges into the beaches may have influenced these results.

The small/absent tidal range and lower wave energy on the shore of many freshwater lakes means sand in these zones can become a microbial sink, developing a microbiological population diversity that differs from that in the water or submerged sediment [59]. A study of two freshwater lake beaches in Canada found higher *E. coli* concentrations in dry sand collected from the foreshore, near the water’s edge, than in sand samples from upshore areas (further from the water) and submerged areas [60].

Finally, extreme weather events can cause FIB concentrations to spike in the upper areas of beaches. The concentrations of enterococci and *E. coli* in sand from the landward edge of a Japanese beach was highest immediately after a typhoon [61]. These bacteria were detected in all layers of the sand, down to 100 cm, but were undetectable one month later. A temporal study of a beach in South Australia detected higher FIB concentrations in sand samples taken during a storm event compared with all other samples taken during a three-month period, particularly in sand samples furthest from the low tide mark [62]. The concentrations had returned to average levels within a week following the storm.

### 4.2. Genetic Indicators for Faecal Contamination

*E. coli* and enterococci have been used to assess the quality of recreational waters for over a century [18]. As explained by Brandão et al. [7], alternative or complementary tests are now available that target genetic material from microorganisms and provide information on faecal source. There are examples of their use for measuring beach sand safety.

Metagenomic Next Generation Sequencing (NGS) is a high-throughput method that amplifies and sequences short pieces of extracted DNA or RNA in a sample. The sequences are compared with a library of genetic data to identify the microorganisms present. The relative abundance of different microorganisms can be calculated. A study applying NGS found that sand samples from recreational beaches in South Africa were dominated by the genera *Bacillus*, *Bifidobacterium* and *Lactobacillus* [63]. Of relevance to considering human health risks, the researchers concluded that non-pathogenic bacteria were most abundant but noted that the presence of probiotic bacteria could indicate the presence of faeces, since these bacteria are naturally found in the intestinal tract of humans and animals. This study shows an advantage of NGS in that it can detect microbes from faecal and non-faecal sources.

Microbial Source Tracking (MST) uses quantitative Polymerase Chain Reaction (qPCR) to target species-specific genetic material from microorganisms or host-bacteria interactions in the gut of animals and humans. Thus, MST is used to identify the sources of faecal contamination. Some markers can additionally indicate aged faecal contamination. For example, the human marker crAssphage has a slower decay rate than FIB and bacterial MST markers, and its continued presence when there are very low concentrations of other human MST markers is considered indicative of aged or treated sewage [20,64]. MST has been used to determine the contamination sources in the supratidal sand of a coastal beach located in Portugal [65]. The work identified that dog faeces were one important source, which helped inform mitigation measures. The presence and concentration of FIB and MST markers for gull faeces has been used to provide evidence of wild birds being an important source of faecal contamination at freshwater beaches in the USA [53,59].

The WHO recreational water quality guidelines note that MST is a useful tool but there is no consistent methodology [1]. WHO recommend multiple lines of evidence before making inferences. It was considered that well-defined methodology and significant knowledge of MST was required to use these tools for tracing faecal contamination, especially where there are multiple faecal sources.

## 5. Microbial Hazards in Beach Sands

Faecal contamination of sand, directly or via faecal-contaminated water, is an important source of many pathogens including zoonotic bacteria (e.g., *Campylobacter* spp. and pathogenic *E. coli*), enteric viruses (e.g., human adenovirus) and zoonotic protozoan parasites (e.g., *Cryptosporidium* spp., *Giardia* spp.). Natural aquatic microbial inhabitants or those that are environmentally widespread, which are also potential human pathogens, can enter beach environments (e.g., *Vibrio* spp., *Pseudomonas aeruginosa*, pathogenic fungi). *Staphylococcus aureus* is an example of an opportunistic pathogen that can be carried into the beach environment by people and subsequently spread into the sand. All these microbes, plus others selected based on published literature, public health surveillance and geography have been profiled in this section as potential health hazards in beach sand. Relevant pathogens need to have the potential to cause adverse health effects if they are ingested, inhaled, or come into contact with skin, although there is currently limited evidence to link their presence in sand with adverse human health events (Section 6).

**Figure 2:**
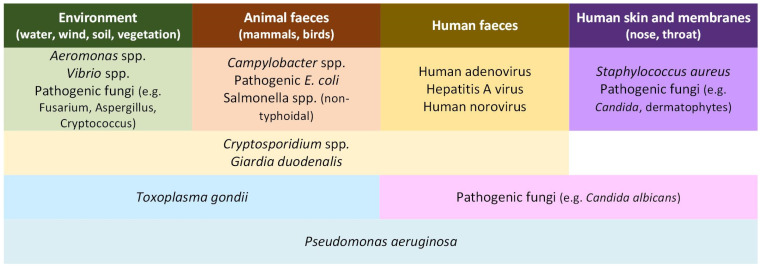
The main reservoirs of microorganisms that are pathogenic to humans entering beach sand environments.

Figure 2 displays the profiled pathogens based on the main reservoirs important for considering beach sand contamination and subsequent human exposure. The categorisation is not definitive, e.g., *Campylobacter* spp., pathogenic *E. coli* and *Salmonella* spp. will be present in faeces from infected people, *S. aureus* can be detected in human faeces [66], *Aeromonas* spp. can be detected in animal faeces [67], and *Cryptosporidium* spp. are excreted with faeces (animals, humans) but recreational exposure to contaminated water is an important cause of human illness [68].

Table 1 summarises information on the abundance and survival of the selected microbiological hazards in beach sand, encompassing some of the information assembled by Whitman et al. [11]. Subsequent sections provide supporting text on each of these hazards and, separately, consider fungal hazards. The information in Table 1 must be considered in the context of each study so has not been numerically standardised to discourage comparisons. In addition to variability introduced through site and method selection, pathogen survival is determined by a range of biotic and abiotic factors, e.g., the type and adaptations of the microorganism itself, the presence of other organisms, nutrients, moisture, temperature, substrate and water movements. In general, pathogenic bacteria and yeasts might multiply outside their hosts if conditions are suitable. Viruses and protozoan parasites require a host to multiply but many survive well in the environment. Generalised information on survival in soils provides some indication of what could be possible in sand at 20–30 °C [69]:

- Bacteria 70 days, usually <20 days (thermotolerant coliforms and *Salmonella* spp.)
- Viruses 70 days, usually <100 days (enterovirus)
- Protozoa 150 days, usually <75 days (*Cryptosporidium* spp.)

While Table 1 suggests the need for survival studies, field studies measuring persistence after a contamination event would be more valuable.

### 5.1. Bacterial Hazards

This section introduces selected bacterial hazards (Table 1) which may be naturally found in beach environments, or which can be introduced directly or indirectly (e.g., via water) from their animal or human reservoir.

*Aeromonas* spp. are ubiquitous, waterborne bacteria [87]. *A. caviae*, *A. veronii*, *A. dhakensis* and *A. hydrophila* are the predominant species isolated from humans with GI [88]. Human infections are known to occur through ingestion of contaminated water or food, or via wound infection while swimming, although the evidence linking exposure and illness is not always robust [89]. Aeromonads have been found in estuarine waters and sediments [90].

*Campylobacter* spp. are commonly found in faeces from wild birds, livestock and companion animals [91,92,93,94], and as faecal contaminants in waterways [64]. *C. jejuni* and *C. coli* are most frequently isolated from human campylobacteriosis cases although other *Campylobacter* species have been reported [95].

Some *E. coli* strains are opportunistic human pathogens which cause enteric or extraintestinal diseases. Pathogenic *E. coli* can be grouped into pathotypes or described using serotypes or virulence genes [96]. Strains of *E. coli* carrying the *stx* genes, named Shiga toxin-producing *E. coli* (STEC), are important causes of intestinal and extraintestinal infections. *E. coli* O157:H7 is an example of an STEC since this serotype commonly carries one or more *stx* genes.

*P. aeruginosa* is an opportunistic human pathogen, known for causing infections in immunocompromised patients and for antimicrobial resistance, so is mainly of concern in healthcare environments [97]. However, this pathogen is also environmentally widespread and known to cause skin and other soft tissue infections through recreational freshwater contact and puncture wounds [98]. *P. aeruginosa* has been investigated as a novel water quality indicator for freshwater beach environments [99].

All serotypes of *Salmonella enterica* subsp. *enterica* are potential human pathogens although some zoonotic serotypes are more commonly isolated from human clinical samples (e.g., Typhimurium, Enteritidis). The focus of this review is on the non-typhoidal salmonellae serotypes, which can be present in the faeces of a range of cold- and warm-blooded animals, particularly that shed by humans and animals experiencing salmonellosis [100]. Humans are the only hosts for typhoidal serotypes Typhi and Paratyphi, which cause enteric fever (typhoid or paratyphoid fever) but can also be chronically shed by asymptomatic carriers [101]. Human sewage is a source of typhoidal salmonellae in regions where enteric fever is endemic [101].

*S. aureus* are common inhabitants of human skin and mucous membranes, being persistently carried by an estimated 20–30% of adults [66]. *S. aureus* also colonise the intestine and have been detected in raw sewage [102], human faeces [66], and faeces from birds (including seagulls) and dogs [103]. Freshwater discharges can contribute to elevated *S. aureus* concentrations in the beach environment [104]. *S. aureus* cause a range of soft tissue, enteric and invasive conditions in humans. The incidence of methicillin-resistant *S. aureus* (MRSA) infections is increasing [81]. In beach environments, the main exposure route presenting a risk of infection is skin contact with *S. aureus* in the sand. *S. aureus* are likely to survive well in beach sand, being tolerant of high temperatures, and dry and salty conditions [103]. *S. aureus* can attach to sand grains, which is likely to support longer persistence, particularly in subsurface sand layers [32]. Higher numbers of *S. aureus* have been found in beach sand during periods of warmer temperatures [80,105], which may reflect that warmer temperatures also attract more visitors to beach areas. The *S. aureus* concentrations in seawater and sand have been positively associated with the density of beach visitors, although not consistently [32,103,106]. Researchers undertaking surveys of *S. aureus* in sand from subtropical beaches in California (USA) proposed that the concentrations (1.87 CFU/g dry sand; 95% CI 0.98–3.90) were not high enough to cause skin/wound infections but admitted more accurate assessment was needed [105]. Much higher concentrations have been reported, e.g., an average concentration of 3.46 × 10^5^ CFU/g dry sand at one beach during a one-year period [32]. It has been suggested that *S. aureus* could be a useful indicator for non-enteric infections [82].

As natural inhabitants of the aquatic environment, *Vibrio* spp. (particularly *V. parahaemolyticus*, *V. vulnificus* and *V. cholerae*) are important hazards for seafood consumers, swimmers and waders [107]. They are known to cause wound infections which can lead to secondary septicaemia. Thus, their presence in wet sand could plausibly lead to wound infections. Because *Vibrio* spp. thrive under warmer conditions, *Vibrio* infections are more common in tropical and subtropical locations compared to temperate locations and there are more sand-related data from these warmer locations (Table 1). *V. alginolyticus*, *V. parahaemolyticus* and *V. vulnificus* have been isolated from beach sand [11,29,85]. The total *Vibrio* spp. count in samples of both backshore and foreshore sand from beaches in Hawaii was as high as 10^2^ CFU/g [54]. *Vibrio* spp. were also among the genera colonising plastic fragments (microplastics) collected from tropical Singapore beaches [108].

Other bacterial pathogens should be briefly mentioned. *Yersinia enterocolitica* and *Yersinia pseudotuberculosis* are important causes of GI (yersiniosis) [109]. None of the reviewed literature suggested that *Yersinia* spp. should be considered in beach environments and no studies of these bacteria in sand were located. However, *Yersinia* spp. share similar characteristics to other zoonotic bacteria included in Table 1, in that they are carried by animals and, via direct faecal contamination or faecal-contaminated water, could enter beach environments. Another cause of GI in humans are *Shigella* spp., which are confined to human hosts and shed in faeces from infected individuals. Shigellosis is more common in regions with poor wastewater infrastructure [110]. It is plausible that beach sand could become contaminated with *Shigella* spp. via human faeces if illness is circulating in the community. *Shigella* spp. were not detected in 130 sand samples from Gaza Beach [111].

### 5.2. Viral Hazards

A range of enteric viruses circulate among humans and might enter beach environments with human faecal contamination and treated (not disinfected) sewage, directly or via water inflows from stormwater or contaminated water bodies. Viruses can be infective in small doses and can remain infectious in the environment for weeks [112,113,114]. Non-enveloped viruses tend to be more environmentally stable because these do not rely on an outer lipid membrane for their survival [115].

Three examples have been considered based on their use in environmental health risk assessment literature and their survival characteristics: Adenovirus, which is a widely used indicator of human faecal contamination, hepatitis A virus and human norovirus, both of which can be found in the environment as a result of human faecal contamination. Data on the presence and survival of viruses in beach sand were scarce so some information from subtropical and tropical locations has been included. Note the infectivity of viruses which are reported by qPCR is unknown as the technique does not distinguish between intact and degraded genetic material.

Adenoviruses are non-enveloped, double-stranded DNA viruses and there are seven species within the genus *Mastadenovirus* that are human host-associated [116]. Most infections are asymptomatic or mild but can progress to severe illness with a range of disease presentations including respiratory, ocular and gastrointestinal symptoms [117]. Adenovirus concentrations in human faeces can reach 10^11^ viral particles/g, and these viruses have been detected in treated wastewater, river water, urban stream water and estuarine water [118].

Hepatitis A virus, now classified as *Hepatovirus ahepa*, is a single-stranded RNA virus that is non-enveloped when excreted with faeces [119]. Three genotypes (GI, GII and GIII) infect humans, resulting in faecal excretions from clinical and subclinical cases that can potentially contain up to 10^11^ GC/g faeces [120]. Hepatitis A virus can persist in the environment for weeks, including in water (fresh, estuarine and seawater) and sediments [112].

Noroviruses are non-enveloped, single-stranded RNA viruses that infect a range of mammalian species, with those infecting humans assigned to the species *Norovirus norwalkense* [121]. Genogroups GI and GII are most often identified from human cases. These cause gastrointestinal infection, resulting in viruses being shed with vomitus and faeces (approximately 10^5^ to 10^9^ GC/g faeces) [122]. Mostly due to prevalence in the community, norovirus is frequently detected in environmental waters [123], but confirming the presence of infectious particles is difficult [124].

### 5.3. Protozoan Parasitic Hazards

Experts have agreed that there are three species of importance for sand safety: *Cryptosporidium* spp., *Giardia duodenalis* and *Toxoplasma gondii* [31]. These protozoan parasites have environmentally persistent life stages (oocysts, cysts) that are excreted with their host’s faeces.

Ingested as an environmentally stable oocyst, *Cryptosporidium* spp. passes through several life-cycle stages within a single host to form new oocysts that are excreted with faeces [125]. Two species cause the majority human infections, the zoonotic species *Cryptosporidium parvum* and the human host-associated species *Cryptosporidium hominis*. *G. duodenalis* (also called *Giardia lamblia* and *Giardia intestinalis*) is a zoonotic pathogen that also completes its life-cycle within a single host, from ingestion of the environmentally stable cyst through to excretion of new cysts with faeces [126]. *Cryptosporidium* spp. and *G. duodenalis* have been detected in livestock faeces and surface waters but may be sensitive to the saline conditions in marine environments [127].

Felines are the definitive host for *T. gondii*. The oocysts are shed with cat faeces then mature into an infectious and environmentally stable form [128]. Toxoplasmosis appearing among marine vertebrates demonstrates that these oocysts migrate into the marine environment and remain infectious [129]. Most human infections are asymptomatic. Toxoplasmosis cases tend to appear sporadically, with pregnant women being a high-risk population, or as part of foodborne or waterborne outbreaks [130]. The risk from beach sand will be elevated in areas with high cat populations.

The potential for sporadic human infection due to metazoan parasites in beach sand should be noted, particularly from the helminths and nematodes which might infect humans via the gastrointestinal tract or skin. It has been proposed that metazoan parasites could be additional indicators for beach hygiene [131]. For example, the helminth *Toxocara* spp., a common parasitic roundworm of domesticated animals, has been of interest because their worldwide distribution [31]. Beach sand surveys have detected *Toxocara* spp. and other parasites [132,133]. The feline hookworm (*Anclyostoma* spp.) has caused an outbreak in a beach setting, due to feral cat overpopulation [1].

### 5.4. Fungal Hazards

Fungi are environmentally widespread and human infections are often opportunistic. Inhaled fungal spores can also cause allergic responses and related conditions such as asthma [17]. People visiting beaches influence the abundance and diversity of sand fungal species. Studies have found positive correlations between the concentration and species of fungi in sand samples and the number of people present in the sampling area [78,106,134,135]. At the beach, adverse health conditions could develop after direct contact with fungal pathogens in the sand or inhalation of disturbed fungal spores.

A variety of fungal genera have been detected in beach sand [17,55]. Potentially pathogenic fungi, such as *Fusarium* spp. and *Aspergillus* spp., could be part of the natural biological community at beaches and harboured in beach wrack [55,136]. Other fungal groups such as yeasts (e.g., *Candida* spp., since reclassified [137]) and dermatophytes are more likely to be associated with the presence of people at the beach [135,136]. FIB appear to be poor indicators for pathogenic fungi, although one study did find correlations between enterococci and yeast concentrations in beach sand [85].

Researchers have considered the fungal species found in beach sand and human clinical samples [55], plus a study of invasive mycotic infections in the San Francisco Bay area during the 1990s [138]. They determined that *Aspergillus*, *Candida*, *Fusarium* and dermatophytes like *Microsporum* and *Trichophyton* represented the sand fungal genera important for human health.

Subsequently, the Mycosands Initiative generated data on fungi in beach sands and waters of both coastal and freshwater bathing sites [17]. Overall, by comparing the geographical regions and individual beaches, the authors found that the presence and composition of fungal microbiota was site-dependent, although *Rhodotorula* and *Candida* species were ubiquitous. The genera most frequently found were *Aspergillus* spp., *Candida* spp., *Fusarium* spp. and *Cryptococcus* spp. Considering all 372 dry sand samples from 91 beaches located in 13 countries, the median concentration of culturable fungi in sand was 89 CFU/g and the maximum 6400 CFU/g. Fungal concentrations in sand were higher than water samples (median 0 CFU/mL, maximum 1592 CFU/mL), although these results are not directly comparable. It was also found that the fungal concentrations were higher in inland freshwater beach sands (although there were fewer samples compared to coastal beach sands), not significantly different between urban and non-urban beaches (although human-associated species more likely to be found in urban beaches), and significantly higher in samples taken during autumn/winter compared with spring/summer (statistically significant, negative correlations were found between the hours of sunshine on the sampling day and the concentrations of total fungi, *Aspergillus* spp. and *Candida* spp.).

Drawing from these studies, the WHO lists eight fungal groups that could be important for sand safety [1]: *Aspergillus* spp., *Cryptococcus* spp., *Histoplasma capsulatum*, *Blastomyces dermatitidis*, *Fusarium* spp., *Cladophialophora bantiana*, *Candida* spp. and the dermatophytes. Of these, infection through inhalation is important for the first five listed. *Candida* spp. are opportunistic pathogens frequently found in sand [55]. One species, *Candida albicans*, is a human gastrointestinal tract commensal so the presence of this species in the environment indicates contamination with human faeces. Dermatophytes are moulds that cause superficial infections of the skin, hair or nails, e.g., *Trichophyton* spp. and *Microsporum* spp. [55].

However, it was acknowledged that there was a lack of data to underpin risk-based decision making, such as dose response and epidemiological data [1,17,55]. Cases can be sporadic and symptoms can be delayed, making it difficult to associate beach sand exposure with illness [139].

## 6. Adverse Health Events Linked to Contact with Beach Sand

Transfer of sand from hand-to-mouth occurs as beach visitors eat, drink, and play in the foreshore and intertidal zones [140]. Studies have shown that children have greater active contact with the sand compared to teenagers and adults, digging and being buried in sand [141,142]. However, age was not important when respondents were asked about eating or drinking after playing in sand, nor about washing their hands after playing in sand. Over half of the respondents reported consuming food/drink and approximately 40% reported washing hands (although the method of washing was not described) [141]. A laboratory study has demonstrated that *E. coli* naturally present in beach sand or F+ coliphage (MS2) (a potential faecal indicator of viruses) added to sand could both be transferred to hands [21]. Hand rinsing removed most of the *E. coli* (86% or more) and coliphage (96% or more), showing that rinsing hands under clean water effectively reduces the risk of pathogen ingestion along the sand-hand-mouth pathway.

Wound infections might also occur from exposure to pathogenic microorganisms in sand. Infection can occur through recent wounds and through wounds occurring while at the beach. It is common for children to visit beaches with existing abrasions and acquire new abrasions while at the beach [143,144].

### 6.1. Epidemiological Studies

Of most value to assessing the risk posed by sand are studies that record behaviours specifically associated with beach sand and water contact, and whether visitors experienced any adverse health effects on the beach day or during subsequent weeks. One problem is that most recreational beach visitors have contact with the water (wading, swimming) as well as the sand, so if they experience adverse health effects it can be difficult to determine whether this occurred from exposure to water or sand. This problem was evident in a systematic review and meta-analysis that found relationships between beach sand contact and adverse health effects were non-significant or inconclusive [145].

A subset of a beach study population can be non-swimmers and their data can be compared to that of swimmers. Over a period of nearly 10 years, data collected through the US National Epidemiological and Environmental Assessment of Recreational Water (NEEAR) study were used to investigate links between recreational beach exposure and illness manifesting within a 10–12 day period following a beach visit. From 54,250 interviews of visitors to freshwater and marine beaches, it was found that swimmers (16.6%) tended to report new health symptoms more often than non-swimmers (13.5%) [146]. The incidence of illness for non-swimmers was greater than the 5% calculated as a background incidence of GI by the WHO [147]. The adverse health symptoms reported by non-swimmers were GI, respiratory illness, ear problems and rashes. From a sub-set of these data (27,365 interviews), it was found that digging in the sand or being buried in the sand were positively associated with GI and diarrhoea [148]. There was no association between sand contact activities and non-enteric illness (respiratory illness, rash, ear or eye ailments, infected cuts). However, swimmers and non-swimmers were not analysed separately; digging in sand and being buried in sand were also strongly associated with water contact.

To complement data collated from one NEEAR cohort (4999 people), 144 wet sand samples were collected from two marine beaches and tested for faecal indicators [149]. Unfortunately, the numbers of participants who did not swim, but who dug in sand or were buried in sand, were low (257 and 24, respectively). This meant that the number of reported health effects was too low among the non-swimmer group to enable comparisons with swimmers. However, there was an increased risk of illness for those who reported digging in sand containing higher enterococci and Bacteroidales concentrations, and people who reported getting sand in their mouth were also more likely to experience GI when the enterococci sand concentrations were higher. As noted previously, there is a stronger correlation between the concentration of enterococci and the incidence of GI when human sewage is the source of faecal contamination at the location of exposure [6].

Further studies have reported that beach visitors who swam were more likely to have adverse health effects compared to non-swimmers [150,151], but sand contact behaviours between these groups were not investigated. Digging in the sand was not associated with norovirus infection among a combined group of swimmers and non-swimmers [150]. As part of a USA study of *S. aureus* in Florida beaches, 18% of 882 beachgoers reported skin conditions within four days of visiting the beach compared to 11% of 609 non-beachgoers (the statistical significance is not reported) [32]. *S. aureus* were detected in relatively high concentrations in the dry sand areas at some visited beaches. The use of control groups who are not beachgoers has been criticised by some, who question whether the overall health status of beachgoers and non-beachgoers should be considered equivalent [27].

The above studies focus on the health of beach visitors. However, the authors of a 2014 review point out that the health of workers who are in frequent contact with sand requires special consideration [31]. No relevant studies were located.

### 6.2. Outbreak Reports

Two outbreak reports were located where exposure to sand was a potential cause of microbiological infection but neither confirmed sand contact as a risk factor. A 2013 outbreak of cryptosporidiosis in the city of Halle (Germany) involved 127 cases and 40 secondary cases who had visited playgrounds, picnic areas and a beach adjacent to the city’s main river [152]. The outbreak began six weeks after the peak of an extensive river flooding event that damaged sewage systems. While oocysts were found in water samples, samples from the beach were not tested. An outbreak of *E. coli* O157 infection in the UK involved cases who had all occupied the same part of a Devon beach on the same day [74]. Foodborne transmission was eliminated and contact with the beach environment was the only plausible risk factor, but *E. coli* O157 were not detected in sand or seawater samples.

Five additional beach outbreak reports were located that were of less relevance to this review but demonstrate the potential for beach visitors to develop illness through contact with this environment. An outbreak of murine typhus among beach sunbathers in Athens, Greece, was probably caused by rats attracted to litter on the beach, which allowed fleas carrying *Rickettsia typhi* to temporarily migrate into the sand [153]. Feline hookworm (*Anclyostoma* spp.) caused an outbreak in a beach setting, due to feral cat overpopulation [1]. An outbreak of cryptococcosis was reported among humans and animals who were exposed to the causative yeast, *Cryptococcus gattii*, in the coastal environment of Vancouver Island, Canada [154]. Genomically linked yeast isolates were found in the coastal forests showing airborne transmission was the likely pathway of infection rather than contact with sand, although beach sand samples were not tested. A fourth report details an outbreak of viral skin infections among beach volleyball athletes [155]. However, the causative human-associated viruses (herpes simplex, molluscum contagiosum and human papilloma) could have been spread through skin contact, wet sand and/or volleyballs, and the researchers did not test sand nor investigate athlete hygiene and shared indoor facilities. A chemical hazard was the more likely cause of the fifth outbreak, occurring during 2019 and involving 29 children and one adult who developed macular erythematous pruritic skin rash two days after sifting sand at the Portuguese island of Azores [13]. There was evidence of faecal contamination, but the causative hazard was most likely elevated levels of sodium hypochlorite due to failed sewage infrastructure.

Outbreaks that have occurred from exposure to sand in non-beach conditions provide evidence to show the potential for illness through active sand contact, without the complication of potential exposure to contaminated water. Salmonellosis was linked to contact with playground sand in Australia, Spain and the Netherlands [156,157,158]. Contamination with faecal matter from wildlife was the confirmed cause of the Australian outbreak, and nesting birds were the probable source for the outbreak in Spain. Sources of contamination were not investigated in the Netherlands study, which used a case–control study to investigate salmonellosis risk factors.

### 6.3. Quantitative Microbial Risk Assessments (QMRAs)

Most QMRAs investigating the risks of illness from pathogenic microorganisms at the beach focus on waterborne exposure [139]. One QMRA has considered exposure to pathogenic microorganisms in sand via oral and dermal pathways [159]. The hand-to-mouth pathway considers the exposure duration and either a single value ingestion rate (g/h), or ingestion calculated from values for sand to skin adherence, hand-to-mouth frequency, the surface area of the skin mouthed and transfer efficiency from hand to mouth. The dermal pathway considers either an exposure scenario when only some contacted sand remains on the skin, or when all the sand remains on the skin and is evenly distributed. The QMRA model was used to calculate the concentration of *Cryptosporidium* spp., enterovirus or *S. aureus* in sand that would be necessary to result in a risk of 1.9 × 10^−2^ illnesses per visit (the USEPA’s 1986 acceptable level of GI among swimmers in marine recreational waters [5]). Considering the different equations applied, and illness risks at the 25th and 50th percentiles, the following concentrations were reported to result in a risk of 19 illnesses per 1000 visits:

- 10–1000 oocysts/g sand of *Cryptosporidium* spp. via oral exposure.
- 5–500 MPN/g sand of enterovirus via oral exposure.
- 10^6^–10^7^ CFU/g sand of *S. aureus* via dermal exposure.

The measured sand concentrations of these pathogens were much lower than the above values, e.g., up to 0.12 oocysts/g for *Cryptosporidium* spp. While this suggested the risk from sand was low, the authors pointed out that faecal contamination will raise sand pathogen concentrations, potentially to the above levels. For *pica* children who consume non-food items, the sand concentrations of *Cryptosporidium* spp. or enterovirus per gram of sand were predicted to be very low to reach the benchmark risk level (2–3 oocysts/g or 1 MPN/g, respectively). The authors also note that the dose response relationship used for *S. aureus* was for intact skin. This risk of infection is likely to be higher for individuals with cuts or wounds. Overall, the outputs are considered conservative, since the QMRA uses infection rates as a proxy for illness rates.

Data for fungal pathogens are currently insufficient to develop a QMRA for beach sand exposure [139]. An important data gap is dose response. Infections by dermatophytes might be common but these rarely lead to severe illness [160]. Manifestation of severe, invasive disease from other pathogenic fungi is host-dependant with the susceptible population being individuals with impaired immunity [160]. Suitable data are not available to calculate the probabilities of infection or illness [1,17,55].

## 7. Integrating Beach Sand Monitoring for Improving Environmental Health

An important limitation to setting risk-based health guidelines for beach sands is the lack of epidemiological evidence linking sand contact with adverse health effects in humans. Epidemiological studies incorporating sand microbiological analyses have been encouraged [7,55]. There is an opportunity to enhance such studies by including cohorts with high beach sand exposure, such those with beach-based occupations (e.g., lifeguards) or who participate in beach sand-based sports (e.g., beach volleyball).

Even without such studies, routine beach sand monitoring might be adopted to proactively establish a baseline of the microbiological beach sand profile (and thus enable deviations to be detected), to measure the impact of activities intended to improve beach conditions, to test the applicability of locally relevant microbiological guideline values, or for other purposes such as addressing data gaps. Proactive sand monitoring along beaches where faecal contamination incidents are known to occur (e.g., during high rainfall events) provides the opportunity to analyse paired sand/water samples for the same microbiological targets. This provides information on what microbiological numbers can be expected in the sand relative to the water and can assist with understanding any ongoing risks from a contamination event after the water quality criteria may have returned to acceptable levels. The persistence of selected pathogens in the sand could also be measured, filling an important data gap. Data from these activities could be integrated with outputs from disease reporting systems to generate regionally relevant hypotheses about the link between beach sand exposure and human illness, supporting future epidemiological studies.

Recommendations from a 2014 meeting of international experts encouraged routine monitoring to be informed by a sanitary survey of the beach environment, considering point and non-point sources of pollution [55]. They also suggested a tiered approach to sand testing. For detecting faecal contamination, the sand would first be tested for FIB concentrations, then MST methods applied to investigate faecal contamination sources, then a third tier focused on testing for specific aetiological agents of disease. They additionally recommended including total culturable fungi in the first tier and fungal identification in the second. This review confirms the practicality of this approach for addressing concerns over known or suspected faecal contamination, particularly since tiers two and three help determine whether there is a health risk or if elevated FIB numbers arose from environmental growth (e.g., due to the presence of seaweed biomass).

There is still uncertainty over the inclusion of microbiological markers to investigate risks from non-faecal associated pathogens in sand. Fungi (total, or some specific groups) and *S. aureus* could be useful indicators of sand microbiological quality. However, the presence and concentration of these microbes in sand cannot yet be linked to infection risk, which makes it difficult to assess the actual risk and set microbiological guideline values. Portugal has adopted guidance values for total fungi in sand as one of their sand safety indicators [1,7]. Under this programme, 80% of samples must contain a total fungal count of ≤490 CFU/g, and a mean guidance value of 89 CFU/g is used as an indicator of beach sand safety. In general, the global spread of fungal pathogens such as *Candida* spp. and *Cryptococcus* spp. poses a significant threat to human health because they produce large quantities of infectious spores in the environment and there are no vaccines and limited antifungal treatments [161].

Finally, it has been recommended that QMRA models incorporate emerging and changing conditions linked with climate change [140]. As discussed by others [140,162], increased temperatures will not necessarily favour microbiological pathogens but are likely to extend the range of those currently more abundant in tropical and subtropical zones, and increased solar irradiation is likely to be detrimental to microbes in the sand surface. In areas experiencing increased precipitation and/or frequency of severe weather events, the entry and survival of pathogenic microorganisms (and supporting nutrients) into the beach environment is likely to increase. Severe weather events can also lead to significant beach erosion, which can remove contaminated sand [163], while rebuilding sandy beaches may introduce pathogens depending on where the sand is sourced. Exposure to beach sand will also change through alterations in human behaviour. People may visit recreational beaches for longer periods during the year as warm periods are extended, although the length and timing of these visits might change, e.g., as people seek to avoid very hot weather.

## 8. Conclusions

Microbiological pathogens in beach sand are a potential health risk for beach visitors, but there is limited epidemiological evidence and important data gaps that hinder risk assessment. Despite this, integrating beach sand testing into recreational water safety programmes and contamination event investigations will proactively generate data to assess the impact of risk management activities. This may support the use of health notices advising the public to avoid sand contact for a period of time after a contamination event. Studies on the health of workers frequently in contact with sand would also create valuable insights. The WHO recommend enterococci as an indicator of the potential presence of faecal-associated pathogens in marine and fresh waters, and in beach sand. *E. coli* might be considered for indicating faecal contamination of beach sands where there is likely to be significant non-human faecal contamination or where there is better alignment with water safety monitoring programmes. A caveat is that both FIB indicate elevated risk from fresh faecal contamination but do not inform on aged sources or non-faecal pathogens. Routine inclusion of non-faecal pathogens into monitoring programmes needs to be justified through evidence from epidemiological studies and human health risk assessment. Complementary tools, including sanitary surveys and molecular test methods, provide a more complete body of evidence to assess human health risk.

## Figures and Tables

**Table 1:** The presence and survival of selected microbiological hazards that could cause human infection and illness via exposure to beach sand.

Microbiological Agent	Most Likely Acute Conditions from Sand Exposure	Presence in Beach Sand *	Survival in Beach Sand
Bacteria			
*Aeromonas* spp.	GI, wound infection	Canada: Detected in interstitial pore water of freshwater beach sand [70].	No relevant data located.
*Campylobacter* spp.	GI	England: Detected in dry and wet beach sand (82/182 positive), including from beaches complying with FIB water standards [71]. USA: Detected in sand (7/53 positive) from coastal beaches [40]. Canada: Detected in pore water from freshwater sand beaches [72].	Concentration decreased in marine beach sand or freshwater beach sand seeded with sewage [40,73].
Pathogenic *E. coli*	GI	England: *E. coli* O157 not detected in 30 sand samples taken from one beach as part of an outbreak investigation [74]. USA: Viable *E. coli* recovered from coastal beach sand but *E. coli* O157:H7 were not identified through PCR-based methods [75]. Virulence genes associated with pathogenic *E. coli* have been detected in sand [11,76].	*E. coli* O157:H7 survived five days in sand in the presence of cattle faeces, both under dry conditions and with seawater tidal simulation [77].
*P. aeruginosa*	Soft tissue infection	USA: Detected in beach sand from temperate South Carolina [78]. Southern Brazil: Detected in marine beach sand [79]. Japan: Non-speciated *Pseudomonas* detected by PCR methods in sand samples taken after a typhoon event, at different depths [61].	No relevant data located.
*Salmonella* spp. (non-typhoidal)	GI	USA: Detected in sand (6/53 positive) from coastal beaches [40]. England: Detected in dry and wet beach sand (10/182 positive), including from beaches complying with FIB water standards [71]. Southern Brazil: Not detected in marine sand [79].	Concentration decreased in marine beach sand or freshwater beach sand seeded with sewage [40,73].
*S. aureus*	Soft tissue infection	USA: Detected in sand (5/37 positive) from coastal beaches (one beach was MRSA positive) [40]. USA: Detected in wet sand 43/210) from freshwater beaches (MRSA detected in 15/210; methicillin-susceptible *S. aureus* (MSSA) in 28/210) [80]. USA: Detected in dry and swash zone marine sand but not in subtidal sand (MRSA only detected in dry sand) [81].	Growth possible but might be limited by natural predation [82]. Concentration decreased in freshwater beach sand seeded with sewage (MRSA concentration also decreased) [73].
*Vibrio* spp.	Wound infection	Japan (post-typhoon): Detected by PCR in sand collected from the water’s edge but not in landward sand samples [61]. Various countries: Detected in studies of beaches located in tropical and subtropical zones [11].	No relevant data located but likely to survive well in wet sand.
Viruses			
Human adenovirus	GI	Portugal: Detected in sand samples [83,84]. USA: Not detected in tropical/dry beaches [75].	No relevant data located.
Hepatitis A virus	Hepatitis	Portugal: Detected in sand samples [83,84]. USA: Not detected in wet and dry sand from a subtropical beach [29].	No relevant data located.
Human norovirus	GI	Portugal: Detected in sand samples [84]. USA: Not detected in wet and dry sand from a subtropical beach [29].	No relevant data located.
Parasites			
*Cryptosporidium* spp.	GI	USA: Detected by PCR but not microscopy in wet and dry sand from a subtropical beach in one study, detected by microscopy in another study (one intertidal sand sample only, 0.63 oocysts/100 g sand) [29,85].	No relevant data located.
*G. duodenalis*	GI	USA: Not detected by PCR or microscopy in wet and dry sand samples from subtropical beaches [29,85]. Southern Brazil: Detected in dry sand (2/96 positive) during spring and summer months [86].	No relevant data located.
*T. gondii*	Toxoplasmosis	No relevant data located.	No relevant data located.

GI, gastrointestinal illness; * Priority given to studies of beaches located in temperate climate zones.

## Data Availability

No new data were created or analysed in this study. Data sharing is not applicable to this article.

## Abbreviations

The following abbreviations are used in this manuscript:

CFU	Colony forming units
CI	Confidence interval
FIB	Faecal indicator bacteria
GC	Genome copies
GI	Gastrointestinal illness
MST	Molecular source tracking
MPN	Most probable number
NGS	Next generation sequencing
PCR	Polymerase chain reaction
QMRA	Quantitative microbial risk assessment
STEC	Shiga toxin-forming *Escherichia coli*
WGS	Whole genome sequencing
WHO	World Health Organization

## References

[1] WHO (2021). Guidelines on Recreational Water Quality. Volume 1: Coastal and Fresh Waters.

[2] Devane M.L., Moriarty E., Weaver L., Cookson A., Gilpin B. (2020). Fecal indicator bacteria from environmental sources; strategies for identification to improve water quality monitoring. Water Res..

[3] Wright M.E., Solo-Gabriele H.M., Elmir S., Fleming L.E. (2009). Microbial load from animal feces at a recreational beach. Mar. Pollut. Bull..

[4] Ministry for the Environment (2003). Microbiological Water Quality Guidelines for Marine and Freshwater Recreational Areas.

[5] USEPA (2012). Recreational Water Quality Criteria.

[6] Wade T.J., Arnold B.F., Schiff K., Colford J.M., Weisberg S.B., Griffith J.F., Dufour A.P. (2022). Health risks to children from exposure to fecally-contaminated recreational water. PLoS ONE.

[7] Brandão J., Valério E., Weiskerger C., Veríssimo C., Sarioglou K., Novak Babič M., Solo-Gabriele H.M., Sabino R., Rebelo M.T. (2023). Strategies for monitoring microbial life in beach sand for protection of public health. Int. J. Environ. Res. Public Health.

[8] Beck H.E., Zimmermann N.E., McVicar T.R., Vergopolan N., Berg A., Wood E.F. (2018). Present and future Köppen-Geiger climate classification maps at 1-km resolution. Sci. Data.

[9] ESR (2025). Notifiable Diseases in New Zealand: Annual Report 2023.

[10] Lee C.M., Lin T.Y., Lin C.C., Kohbodi G.A., Bhatt A., Lee R., Jay J.A. (2006). Persistence of fecal indicator bacteria in Santa Monica Bay beach sediments. Water Res..

[11] Whitman R.L., Harwood V.J., Edge T.A., Nevers M.B., Byappanahalli M., Vijayavel K., Brandao J., Sadowsky M.J., Alm E.W., Crowe A. (2014). Microbes in beach sands: Integrating environment, ecology and public health. Rev. Environ. Sci. Biotechnol..

[12] Staley C., Sadowsky M.J. (2016). Regional similarities and consistent patterns of local variation in beach sand bacterial communities throughout the Northern Hemisphere. Appl. Environ. Microbiol..

[13] Brandão J., Albergaria I., Albuquerque J., José S., Grossinho J., Ferreira F.C., Raposo A., Rodrigues R., Silva C., Jordao L. (2020). Untreated sewage contamination of beach sand from a leaking underground sewage system. Sci. Total Environ..

[14] De Bhowmick G., Sarmah A.K., Dubey B. (2021). Microplastics in the NZ environment: Current status and future directions. Case Stud. Chem. Environ. Eng..

[15] Hernandez R.J., Hernandez Y., Jimenez N.H., Piggot A.M., Klaus J.S., Feng Z.X., Reniers A., Solo-Gabriele H.M. (2014). Effects of full-scale beach renovation on fecal indicator levels in shoreline sand and water. Water Res..

[16] Archana A., Francis C., Boehm A. (2021). The beach aquifer microbiome: Research gaps and data needs. Front. Environ. Sci..

[17] Brandão J., Gangneux J.P., Arikan-Akdagli S., Barac A., Bostanaru A.C., Brito S., Bull M., Çerikçioğlu N., Chapman B., Efstratiou M.A. (2021). Mycosands: Fungal diversity and abundance in beach sand and recreational waters—Relevance to human health. Sci. Total Environ..

[18] Korajkic A., McMinn B.R., Harwood V.J. (2018). Relationships between microbial indicators and pathogens in recreational water settings. Int. J. Environ. Res. Public Health.

[19] Rodrigues C., Cunha M.Â. (2017). Assessment of the microbiological quality of recreational waters: Indicators and methods. Euro-Mediterr. J. Environ. Integr..

[20] Boehm A.B., Graham K.E., Jennings W.C. (2018). Can we swim yet? Systematic review, meta-analysis, and risk assessment of aging sewage in surface waters. Environ. Sci. Technol..

[21] Whitman R.L., Przybyla-Kelly K., Shively D.A., Nevers M.B., Byappanahalli M.N. (2009). Hand-mouth transfer and potential for exposure to *E. coli* and F+ coliphage in beach sand, Chicago, Illinois. J. Water Health.

[22] Yamahara K.M., Layton B.A., Santoro A.E., Boehm A.B. (2007). Beach sands along the California coast are diffuse sources of fecal bacteria to coastal waters. Environ. Sci. Technol..

[23] Rumball N.A., Alm E.W., McLellan S.L. (2023). Genetic determinants of *Escherichia coli* survival in beach sand. Appl. Environ. Microbiol..

[24] Alm E.W., Burke J., Spain A. (2003). Fecal indicator bacteria are abundant in wet sand at freshwater beaches. Water Res..

[25] Phillips M.C., Solo-Gabriele H.M., Piggot A.M., Klaus J.S., Zhang Y.F. (2011). Relationships between sand and water quality at recreational beaches. Water Res..

[26] Whitman R.L., Nevers M.B. (2003). Foreshore sand as a source of *Escherichia coli* in nearshore water of a Lake Michigan beach. Appl. Environ. Microbiol..

[27] Bonilla T.D., Nowosielski K., Cuvelier M., Hartz A., Green M., Esiobu N., McCorquodale D.S., Fleisher J.M., Rogerson A. (2007). Prevalence and distribution of fecal indicator organisms in South Florida beach sand and preliminary assessment of health effects associated with beach sand exposure. Mar. Pollut. Bull..

[28] Zhang Q., He X., Yan T. (2015). Differential decay of wastewater bacteria and change of microbial communities in beach sand and seawater microcosms. Environ. Sci. Technol..

[29] Abdelzaher A.M., Wright M.E., Ortega C., Solo-Gabriele H.M., Miller G., Elmir S., Newman X., Shih P., Bonilla J.A., Bonilla T.D. (2010). Presence of pathogens and indicator microbes at a non-point source subtropical recreational marine beach. Appl. Environ. Microbiol..

[30] Halliday E., Gast R.J. (2011). Bacteria in beach sands: An emerging challenge in protecting coastal water quality and bather health. Environ. Sci. Technol..

[31] Sabino R., Rodrigues R., Costa I., Carneiro C., Cunha M., Duarte A., Faria N., Ferreira F.C., Gargaté M.J., Júlio C. (2014). Routine screening of harmful microorganisms in beach sands: Implications to public health. Sci. Total Environ..

[32] Esiobu N., Green M., Echeverry A., Bonilla T.D., Stinson C.M., Hartz A., Rogerson A., McCorquodale D.S. (2013). High numbers of *Staphylococcus aureus* at three bathing beaches in South Florida. Int. J. Environ. Res. Public Health.

[33] Gerbersdorf S.U., Koca K., de Beer D., Chennu A., Noss C., Risse-Buhl U., Weitere M., Eiff O., Wagner M., Aberle J. (2020). Exploring flow-biofilm-sediment interactions: Assessment of current status and future challenges. Water Res..

[34] Malham S.K., Rajko-Nenow P., Howlett E., Tuson K.E., Perkins T.L., Pallett D.W., Wang H., Jago C.F., Jones D.L., McDonald J.E. (2014). The interaction of human microbial pathogens, particulate material and nutrients in estuarine environments and their impacts on recreational and shellfish waters. Environ. Sci. Process. Impacts.

[35] Hassard F., Gwyther C.L., Farkas K., Andrews A., Jones V., Cox B., Brett H., Jones D.L., McDonald J.E., Malham S.K. (2016). Abundance and distribution of enteric bacteria and viruses in coastal and estuarine sediments—A review. Front. Microbiol..

[36] Fries J.S., Characklis G.W., Noble R.T. (2008). Sediment-water exchange of *Vibrio* sp. and fecal indicator bacteria: Implications for persistence and transport in the Neuse River Estuary, North Carolina, USA. Water Res..

[37] Piggot A.M., Klaus J.S., Johnson S., Phillips M.C., Solo-Gabriele H.M. (2012). Relationship between enterococcal levels and sediment biofilms at recreational beaches in South Florida. Appl. Environ. Microbiol..

[38] Korajkic A., Wanjugi P., Brooks L., Cao Y., Harwood V.J. (2019). Persistence and decay of fecal microbiota in aquatic habitats. Microbiol. Mol. Biol. Rev..

[39] Yamahara K.M., Walters S.P., Boehm A.B. (2009). Growth of enterococci in unaltered, unseeded beach sands subjected to tidal wetting. Appl. Environ. Microbiol..

[40] Yamahara K.M., Sassoubre L.M., Goodwin K.D., Boehm A.B. (2012). Occurrence and persistence of bacterial pathogens and indicator organisms in beach sand along the California coast. Appl. Environ. Microbiol..

[41] Mika K.B., Imamura G., Chang C., Conway V., Fernandez G., Griffith J.F., Kampalath R.A., Lee C.M., Lin C.-C., Moreno R. (2009). Pilot- and bench-scale testing of faecal indicator bacteria survival in marine beach sand near point sources. J. Appl. Microbiol..

[42] Beversdorf L.J., Bornstein-Forst S.M., McLellan S.L. (2007). The potential for beach sand to serve as a reservoir for *Escherichia coli* and the physical influences on cell die-off. J. Appl. Microbiol..

[43] Abdool-Ghany A.A., Sahwell P.J., Klaus J., Gidley M.L., Sinigalliano C.D., Solo-Gabriele H.M. (2022). Fecal indicator bacteria levels at a marine beach before, during, and after the COVID-19 shutdown period and associations with decomposing seaweed and human presence. Sci. Total Environ..

[44] Halliday E., Ralston D.K., Gast R.J. (2015). Contribution of sand-associated enterococci to dry weather water quality. Environ. Sci. Technol..

[45] Hartz A., Cuvelier M., Nowosielski K., Bonilla T.D., Green M., Esiobu N., McCorquodale D.S., Rogerson A. (2008). Survival potential of *Escherichia coli* and enterococci in subtropical beach sand: Implications for water quality managers. J. Environ. Qual..

[46] Korajkic A., Wanjugi P., Harwood V.J. (2013). Indigenous microbiota and habitat influence *Escherichia coli* survival more than sunlight in simulated aquatic environments. Appl. Environ. Microbiol..

[47] Feng F., Goto D., Yan T. (2010). Effects of autochthonous microbial community on the die-off of fecal indicators in tropical beach sand. FEMS Microbiol. Ecol..

[48] Anderson S.A., Turner S.J., Lewis G.D. (1997). Enterococci in the New Zealand environment: Implications for water quality monitoring. Water Sci. Technol..

[49] Imamura G.J., Thompson R.S., Boehm A.B., Jay J.A. (2011). Wrack promotes the persistence of fecal indicator bacteria in marine sands and seawater. FEMS Microbiol. Ecol..

[50] Kinzelman J.L., Pond K.R., Longmaid K.D., Bagley R.C. (2004). The effect of two mechanical beach grooming strategies on *Escherichia coli* density in beach sand at a southwestern Lake Michigan beach. Aquat. Ecosyst. Health Manag..

[51] Kinzelman J.L., Whitman R.L., Byappanahalli M., Jackson E., Bagley R.C. (2003). Evaluation of beach grooming techniques on *Escherichia coli* density in foreshore sand at North Beach, Racine, WI. Lake Reserv. Manag..

[52] Metcalf R., Fellows R., White H.L., Quilliam R.S. (2024). Persistence of ‘wet wipes’ in beach sand: An unrecognised reservoir for localised *E. coli* contamination. Mar. Pollut. Bull..

[53] Cloutier D.D., McLellan S.L. (2017). Distribution and differential survival of traditional and alternative indicators of fecal pollution at freshwater beaches. Appl. Environ. Microbiol..

[54] Cui H.L., Yang K., Pagaling E., Yan T. (2013). Spatial and temporal variation in enterococcal abundance and its relationship to the microbial community in Hawaii beach sand and water. Appl. Environ. Microbiol..

[55] Solo-Gabriele H.M., Harwood V.J., Kay D., Fujioka R.S., Sadowsky M.J., Whitman R.L., Wither A., Caniça M., Carvalho da Fonseca R., Duarte A. (2016). Beach sand and the potential for infectious disease transmission: Observations and recommendations. J. Mar. Biol. Assoc. UK.

[56] Gast R.J., Elgar S., Raubenheimer B. (2015). Observations of transport of bacterial-like microspheres through beach sand. Cont. Shelf Res..

[57] Feng Z., Reniers A., Haus B.K., Solo-Gabriele H.M., Kelly E.A. (2016). Wave energy level and geographic setting correlate with Florida beach water quality. Mar. Pollut. Bull..

[58] Vogel L.J., O’Carroll D.M., Edge T.A., Robinson C.E. (2016). Release of *Escherichia coli* from foreshore sand and pore water during intensified wave conditions at a recreational beach. Environ. Sci. Technol..

[59] Nevers M.B., Byappanahalli M.N., Nakatsu C.H., Kinzelman J.L., Phanikumar M.S., Shively D.A., Spoljaric A.M. (2020). Interaction of bacterial communities and indicators of water quality in shoreline sand, sediment, and water of Lake Michigan. Water Res..

[60] Staley Z.R., Vogel L., Robinson C., Edge T.A. (2015). Differential occurrence of *Escherichia coli* and human Bacteroidales at two Great Lakes beaches. J. Great Lakes Res..

[61] Suzuki Y., Teranishi K., Matsuwaki T., Nukazawa K., Ogura Y. (2018). Effects of bacterial pollution caused by a strong typhoon event and the restoration of a recreational beach: Transitions of fecal bacterial counts and bacterial flora in beach sand. Sci. Total Environ..

[62] Whiley H., Austin J., da Silva G.M., Ross K. (2018). Faecal indicator bacteria present in sand at South Port Beach, South Australia. J. Coast. Res..

[63] Sibanda T., Ramganesh S. (2021). Taxonomic and functional analyses reveal existence of virulence and antibiotic resistance genes in beach sand bacterial populations. Arch. Microbiol..

[64] Leonard M., Gilpin B., Horn B., Coxon S., Armstrong B., Scholes P., Haysome I., Priya B., Eaton C., Cornelius A. (2021). Quantitative Microbial Risk Assessment Phase 2.1—Initial Data Collection and Recommendations.

[65] Valério E., Santos M.L., Teixeira P., Matias R., Mendonça J., Ahmed W., Brandão J. (2022). Microbial Source Tracking as a method of determination of beach sand contamination. Int. J. Environ. Res. Public Health.

[66] Piewngam P., Otto M. (2024). *Staphylococcus aureus* colonisation and strategies for decolonisation. Lancet Microbe.

[67] Ceylan E., Berktas M., Ağaoğlu Z. (2009). The occurrence and antibiotic resistance of motile *Aeromonas* in livestock. Trop. Anim. Health Prod..

[68] Kooh P., Thébault A., Cadavez V., Gonzales-Barron U., Villena I. (2021). Risk factors for sporadic cryptosporidiosis: A systematic review and meta-analysis. Microb. Risk Anal..

[69] WHO (2006). Guidelines for the Safe Use of Wastewater, Excreta and Greywater.

[70] Khan I.U., Loughborough A., Edge T.A. (2009). DNA-based real-time detection and quantification of aeromonads from fresh water beaches on Lake Ontario. J. Water Health.

[71] Bolton F.J., Surman S.B., Martin K., Wareing D.R., Humphrey T.J. (1999). Presence of *Campylobacter* and *Salmonella* in sand from bathing beaches. Epidemiol. Infect..

[72] Khan I.U.H., Hill S., Nowak E., Palmer M.E., Jarjanazi H., Lee D.Y., Mueller M., Schop R., Weir S., Irwin Abbey A.M. (2013). Investigation of the prevalence of thermophilic *Campylobacter* species at Lake Simcoe recreational beaches. Inland Waters.

[73] Eichmiller J.J., Borchert A.J., Sadowsky M.J., Hicks R.E. (2014). Decay of genetic markers for fecal bacterial indicators and pathogens in sand from Lake Superior. Water Res..

[74] Harrison S., Kinra S. (2004). Outbreak of *Escherichia coli* O157 associated with a busy bathing beach. Commun. Dis. Public Health.

[75] Goodwin K.D., Matragrano L., Wanless D., Sinigalliano C.D., LaGier M.J. (2009). A preliminary investigation of fecal indicator bacteria, human pathogens, and source tracking markers in beach water and sand. Environ. Res. J..

[76] Cabot M.E., Piccini C., Inchausti P., de la Escalera G.M., García-Alonso J. (2024). Relationships between fecal indicator abundance in water and sand and the presence of pathogenic genes in sand of recreational beaches. Environ. Monit. Assess..

[77] Williams A.P., Avery L.M., Killham K., Jones D.L. (2007). Persistence, dissipation, and activity of *Escherichia coli* O157:H7 within sand and seawater environments. FEMS Microbiol. Ecol..

[78] Stevens J., Evans G., Aguirre K. (2012). Human beach use affects abundance and identity of fungi present in sand. J. Coast. Res..

[79] Sanchez P.S., Agudo E.G., Castro F.G., Alves M.N., Martins M.T. (1986). Evaluation of the sanitary quality of marine recreational waters and sands from beaches of the São Paulo State, Brazil. Water Sci. Technol..

[80] Thapaliya D., Hellwig E.J., Kadariya J., Grenier D., Jefferson A.J., Dalman M., Kennedy K., DiPerna M., Orihill A., Taha M. (2017). Prevalence and characterization of *Staphylococcus aureus* and methicillin-resistant *Staphylococcus aureus* on public recreational beaches in Northeast Ohio. Geohealth.

[81] Plano L.R., Shibata T., Garza A.C., Kish J., Fleisher J.M., Sinigalliano C.D., Gidley M.L., Withum K., Elmir S.M., Hower S. (2013). Human-associated methicillin-resistant *Staphylococcus aureus* from a subtropical recreational marine beach. Microb. Ecol..

[82] Mohammed R.L., Echeverry A., Stinson C.M., Green M., Bonilla T.D., Hartz A., McCorquodale D.S., Rogerson A., Esiobu N. (2012). Survival trends of *Staphylococcus aureus*, *Pseudomonas aeruginosa*, and *Clostridium perfringens* in a sandy South Florida beach. Mar. Pollut. Bull..

[83] Monteiro S., Brondani G., Brandão J., Santos R. Viruses in beach sand. Proceedings of the Fifth Food and Environmental Virology Congress.

[84] Robalo A., Brandão J., Shibata T., Solo-Gabriele H., Santos R., Monteiro S. (2023). Detection of enteric viruses and SARS-CoV-2 in beach sand. Sci. Total Environ..

[85] Shah A.H., Abdelzaher A.M., Phillips M., Hernandez R., Solo-Gabriele H.M., Kish J., Scorzetti G., Fell J.W., Diaz M.R., Scott T.M. (2011). Indicator microbes correlate with pathogenic bacteria, yeasts and helminthes in sand at a subtropical recreational beach site. J. Appl. Microbiol..

[86] Zanoli Sato M., Di Bari M., Lamparelli C., Truzzi A., Coelho M., Hachich E. (2005). Sanitary quality of sands from marine recreational beaches of São Paulo, Brazil. Braz. J. Microbiol..

[87] Tomás J.M. (2012). The main *Aeromonas* pathogenic factors. ISRN Microbiol..

[88] Fernández-Bravo A., Figueras M.J. (2020). An update on the genus *Aeromonas*: Taxonomy, epidemiology, and pathogenicity. Microorganisms.

[89] USFDA (2012). Bad Bug Book. Handbook of Foodborne Pathogenic Microorganisms and Natural Toxins.

[90] Chaix G., Roger F., Berthe T., Lamy B., Jumas-Bilak E., Lafite R., Forget-Leray J., Petit F. (2017). Distinct *Aeromonas* populations in water column and associated with copepods from estuarine environment (Seine, France). Front. Microbiol..

[91] Mohan V. (2015). Faeco-prevalence of *Campylobacter jejuni* in urban wild birds and pets in New Zealand. BMC Res. Notes.

[92] Moriarty E.M., McEwan N., Mackenzie M., Karki N., Sinton L.W. (2011). Incidence and prevalence of microbial indicators and pathogens in ovine faeces in New Zealand. N. Z. J. Agric. Res..

[93] Moriarty E.M., Karki N., Mackenzie M., Sinton L.W., Wood D.R., Gilpin B.J. (2011). Faecal indicators and pathogens in selected New Zealand waterfowl. N. Z. J. Mar. Freshw. Res..

[94] Rapp D., Ross C.M., Pleydell E.J., Muirhead R.W. (2012). Differences in the fecal concentrations and genetic diversities of *Campylobacter jejuni* populations among individual cows in two dairy herds. Appl. Environ. Microbiol..

[95] Facciolà A., Riso R., Avventuroso E., Visalli G., Delia S.A., Laganà P. (2017). *Campylobacter*: From microbiology to prevention. J. Prev. Med. Hyg..

[96] Pakbin B., Brück W.M., Rossen J.W.A. (2021). Virulence factors of enteric pathogenic *Escherichia coli*: A review. Int. J. Mol. Sci..

[97] Silby M.W., Winstanley C., Godfrey S.A.C., Levy S.B., Jackson R.W. (2011). *Pseudomonas* genomes: Diverse and adaptable. FEMS Microbiol. Rev..

[98] Wilson M.G., Pandey S. (2022). Pseudomonas aeruginosa. StatPearls.

[99] Esiobu N., Mohammed R., Echeverry A., Green M., Bonilla T., Hartz A., McCorquodale D., Rogerson A. (2004). The application of peptide nucleic acid probes for rapid detection and enumeration of eubacteria, *Staphylococcus aureus* and *Pseudomonas aeruginosa* in recreational beaches of S. Florida. J. Microbiol. Methods.

[100] Jajere S.M. (2019). A review of *Salmonella enterica* with particular focus on the pathogenicity and virulence factors, host specificity and antimicrobial resistance including multidrug resistance. Vet. World.

[101] Crump J.A. (2019). Progress in typhoid fever epidemiology. Clin. Infect. Dis..

[102] Kozajda A., Jeżak K. (2020). Occupational exposure to *Staphylococcus aureus* in the wastewater treatment plants environment. Med. Pr..

[103] Topić N., Cenov A., Jozić S., Glad M., Mance D., Lušić D., Kapetanović D., Mance D., Vukić Lušić D. (2021). *Staphylococcus aureus*—An additional parameter of bathing water quality for crowded urban beaches. Int. J. Environ. Res. Public Health.

[104] Steadmon M., Takakusagi M., Wiegner T.N., Jones M., Economy L.M., Panelo J., Morrison L.A., Medeiros M.C.I., Frank K.L. (2024). Detection and modeling of *Staphylococcus aureus* and fecal bacteria in Hawaiian coastal waters and sands. Water Environ. Res..

[105] Goodwin K.D., McNay M., Cao Y.P., Ebentier D., Madison M., Griffith J.F. (2012). A multi-beach study of *Staphylococcus aureus*, MRSA, and enterococci in seawater and beach sand. Water Res..

[106] Papadakis J.A., Mavridou A., Richardson S.C., Lampiri M., Marcelou U. (1997). Bather-related microbial and yeast populations in sand and seawater. Water Res..

[107] Baker-Austin C., Oliver J.D., Alam M., Ali A., Waldor M.K., Qadri F., Martinez-Urtaza J. (2018). *Vibrio* spp. infections. Nat. Rev. Dis. Primers.

[108] Curren E., Leong S.C.Y. (2019). Profiles of bacterial assemblages from microplastics of tropical coastal environments. Sci. Total Environ..

[109] Cressey P., Horn B., Gilpin B., Rivas L. (2025). The burden of yersiniosis in New Zealand, 2022. N. Z. Med. J..

[110] Aslam A., Hashmi M., Okafor C. (2024). Shigellosis. StatPearls [Internet].

[111] Elmanama A.A., Fahd M.I., Afifi S., Abdallah S., Bahr S. (2005). Microbiological beach sand quality in Gaza Strip in comparison to seawater quality. Environ. Res..

[112] Di Cola G., Fantilli A.C., Pisano M.B., Ré V.E. (2021). Foodborne transmission of hepatitis A and hepatitis E viruses: A literature review. Int. J. Food Microbiol..

[113] Rexin D., Rachmadi A.T., Hewitt J. (2024). Persistence of infectious human norovirus in estuarine water. Food Environ. Virol..

[114] Teunis P.F.M., Le Guyader F.S., Liu P., Ollivier J., Moe C.L. (2020). Noroviruses are highly infectious but there is strong variation in host susceptibility and virus pathogenicity. Epidemics.

[115] Firquet S., Beaujard S., Lobert P.E., Sané F., Caloone D., Izard D., Hober D. (2015). Survival of enveloped and non-enveloped viruses on inanimate surfaces. Microbes Environ..

[116] Benkő M., Aoki K., Arnberg N., Davison A.J., Echavarría M., Hess M., Jones M.S., Kaján G.L., Kajon A.E., Mittal S.K. (2022). ICTV virus taxonomy profile: Adenoviridae 2022. J. Gen. Virol..

[117] Khanal S., Ghimire P., Dhamoon A.S. (2018). The repertoire of adenovirus in human disease: The innocuous to the deadly. Biomedicines.

[118] Hewitt J., Greening G.E., Leonard M., Lewis G.D. (2013). Evaluation of human adenovirus and human polyomavirus as indicators of human sewage contamination in the aquatic environment. Water Res..

[119] Zell R., Delwart E., Gorbalenya A.E., Hovi T., King A.M.Q., Knowles N.J., Lindberg A.M., Pallansch M.A., Palmenberg A.C., Reuter G. (2017). ICTV virus taxonomy profile: Picornaviridae. J. Gen. Virol..

[120] Sayed I.M. (2023). Dual infection of hepatitis A virus and hepatitis E virus—What Is known?. Viruses.

[121] Vinjé J., Estes M.K., Esteves P., Green K.Y., Katayama K., Knowles N.J., L’Homme Y., Martella V., Vennema H., White P.A. (2019). ICTV virus taxonomy profile: Caliciviridae. J. Gen. Virol..

[122] Teunis P.F., Sukhrie F.H., Vennema H., Bogerman J., Beersma M.F., Koopmans M.P. (2015). Shedding of norovirus in symptomatic and asymptomatic infections. Epidemiol. Infect..

[123] Ekundayo T.C., Igere B.E., Oluwafemi Y.D., Iwu C.D., Olaniyi O.O. (2021). Human norovirus contamination in water sources: A systematic review and meta-analysis. Environ. Pollut..

[124] Chandran S., Gibson K.E. (2024). Improving the detection and understanding of infectious human norovirus in food and water matrices: A review of methods and emerging models. Viruses.

[125] Vanathy K., Parija S.C., Mandal J., Hamide A., Krishnamurthy S. (2017). Cryptosporidiosis: A mini review. Trop. Parasitol..

[126] Adam R.D. (2001). Biology of *Giardia lamblia*. Clin. Microbiol. Rev..

[127] Johnson D.C., Enriquez C.E., Pepper I.L., Davis T.L., Gerba C.P., Rose J.B. (1997). Survival of *Giardia*, *Cryptosporidium*, Poliovirus and *Salmonella* in marine waters. Water Sci. Technol..

[128] Shapiro K., Bahia-Oliveira L., Dixon B., Dumètre A., de Wit L.A., VanWormer E., Villena I. (2019). Environmental transmission of *Toxoplasma gondii*: Oocysts in water, soil and food. Food Waterborne Parasitol..

[129] Roberts J.O., Jones H.F.E., Roe W.D. (2021). The effects of *Toxoplasma gondii* on New Zealand wildlife: Implications for conservation and management. Pac. Conserv. Biol..

[130] Pinto-Ferreira F., Caldart E.T., Pasquali A.K.S., Mitsuka-Breganó R., Freire R.L., Navarro I.T. (2019). Patterns of transmission and sources of infection in outbreaks of human toxoplasmosis. Emerg. Infect. Dis..

[131] Manjarrez G., Blanco J., Gonzalez B., Botero C.M., Diaz-Mendoza C. (2019). Parasites in tourist beaches: Proposal for its inclusion as health quality indicators. Review for Latin America. Ecol. Apl..

[132] Bojar H., Kłapeć T. (2018). Contamination of selected recreational areas in Lublin Province, Eastern Poland, by eggs of *Toxocara* spp., *Ancylostoma* spp. and *Trichuris* spp.. Ann. Agric. Environ. Med..

[133] Ramos E.L.P., Gómez-Hernández C., Queiroz L.G., Moura R.G.F., Nogueira N.P., Ferreira G.L.S., Rezende-Oliveira K. (2020). Parasite detection in sand from bays on the north coast of São Paulo state, Brazil. J. Trop. Pathol..

[134] Vogel C., Rogerson A., Schatz S., Laubach H., Tallman A., Fell J. (2007). Prevalence of yeasts in beach sand at three bathing beaches in South Florida. Water Res..

[135] Deligios M., Mazzarello V., Fiamma M., Barac A., Diana L., Ferrari M., Murgia M., Paglietti B., Rubino S. (2023). Seasonal variation in fungi in beach sand in summertime: Stintino (Italy). Int. J. Environ. Res. Public Health.

[136] Abreu R., Figueira C., Romão D., Brandão J., Freitas M.C., Andrade C., Calado G., Ferreira C., Campos A., Prada S. (2016). Sediment characteristics and microbiological contamination of beach sand—A case-study in the archipelago of Madeira. Sci. Total Environ..

[137] Liu F., Hu Z.D., Zhao X.M., Zhao W.N., Feng Z.X., Yurkov A., Alwasel S., Boekhout T., Bensch K., Hui F.L. (2024). Phylogenomic analysis of the *Candida auris*-*Candida haemuli* clade and related taxa in the *Metschnikowiaceae*, and proposal of thirteen new genera, fifty-five new combinations and nine new species. Persoonia.

[138] Rees J.R., Pinner R.W., Hajjeh R.A., Brandt M.E., Reingold A.L. (1998). The epidemiological features of invasive mycotic infections in the San Francisco Bay area, 1992-1993: Results of population-based laboratory active surveillance. Clin. Infect. Dis..

[139] Weiskerger C.J., Brandão J. (2020). Fungal contaminants in water and sand: A new frontier for quantitative microbial risk assessment. Curr. Opin. Environ. Sci. Health.

[140] Brandão J., Weiskerger C., Valério E., Pitkänen T., Meriläinen P., Avolio L., Heaney C.D., Sadowsky M.J. (2022). Climate change impacts on microbiota in beach sand and water: Looking ahead. Int. J. Environ. Res. Public Health.

[141] DeFlorio-Barker S., Arnold B.F., Sams E.A., Dufour A.P., Colford J.M., Weisberg S.B., Schiff K.C., Wade T.J. (2018). Child environmental exposures to water and sand at the beach: Findings from studies of over 68,000 subjects at 12 beaches. J. Expo. Sci. Environ. Epidemiol..

[142] Ferguson A., Del Donno C., Obeng-Gyasi E., Mena K., Kaur Altomare T., Guerrero R., Gidley M., Montas L., Solo-Gabriele H.M. (2019). Children exposure-related behavior patterns and risk perception associated with recreational beach use. Int. J. Environ. Res. Public Health.

[143] Moran K., Webber J. (2014). Surf, sand, scrapes and stings: First aid incidents involving children at New Zealand beaches, 2007–2012. J. Paediatr. Child Health.

[144] Tomenchok L.E., Gidley M.L., Mena K.D., Ferguson A.C., Solo-Gabriele H.M. (2020). Children’s abrasions in recreational beach areas and a review of possible wound infections. Int. J. Environ. Res. Public Health.

[145] Russo G.S., Eftim S.E., Goldstone A.E., Dufour A.P., Nappier S.P., Wade T.J. (2020). Evaluating health risks associated with exposure to ambient surface waters during recreational activities: A systematic review and meta-analysis. Water Res..

[146] Collier S.A., Wade T.J., Sams E.A., Hlavsa M.C., Dufour A.P., Beach M.J. (2015). Swimming in the USA: Beachgoer characteristics and health outcomes at US marine and freshwater beaches. J. Water Health.

[147] WHO (2003). Guidelines for Safe Recreational Water Environments. Volume 1: Coastal and Freshwaters.

[148] Heaney C.D., Sams E., Wing S., Marshall S., Brenner K., Dufour A.P., Wade T.J. (2009). Contact with beach sand among beachgoers and risk of illness. Am. J. Epidemiol..

[149] Heaney C.D., Sams E., Dufour A.P., Brenner K.P., Haugland R.A., Chern E., Wing S., Marshall S., Love D.C., Serre M. (2012). Fecal indicators in sand, sand contact, and risk of enteric illness among beachgoers. Epidemiology.

[150] Wade T.J., Augustine S.A.J., Griffin S.M., Sams E.A., Oshima K.H., Egorov A.I., Simmons K.J., Eason T.N., Dufour A.P. (2018). Asymptomatic norovirus infection associated with swimming at a tropical beach: A prospective cohort study. PLoS ONE.

[151] Leonard A.F.C., Garside R., Ukoumunne O.C., Gaze W.H. (2020). A cross-sectional study on the prevalence of illness in coastal bathers compared to non-bathers in England and Wales: Findings from the Beach User Health Survey. Water Res..

[152] Gertler M., Dürr M., Renner P., Poppert S., Askar M., Breidenbach J., Frank C., Preußel K., Schielke A., Werber D. (2015). Outbreak of *Cryptosporidium hominis* following river flooding in the city of Halle (Saale), Germany, August 2013. BMC Infect. Dis..

[153] Labropoulou S., Charvalos E., Chatzipanagiotou S., Ioannidis A., Sylignakis P., Τaka S., Karageorgou I., Linou M., Mpizta G., Mentis A. (2021). Sunbathing, a possible risk factor of murine typhus infection in Greece. PLoS Negl. Trop. Dis..

[154] Kidd S.E., Hagen F., Tscharke R.L., Huynh M., Bartlett K.H., Fyfe M., Macdougall L., Boekhout T., Kwon-Chung K.J., Meyer W. (2004). A rare genotype of *Cryptococcus gattii* caused the cryptococcosis outbreak on Vancouver Island (British Columbia, Canada). Proc. Natl. Acad. Sci. USA.

[155] Tertipi N., Kefala V., Papageorgiou E., Rallis E. (2021). Prevalence of common viral skin infections in beach volleyball athletes. Viruses.

[156] Doorduyn Y., Van Den Brandhof W.E., Van Duynhoven Y.T., Wannet W.J., Van Pelt W. (2006). Risk factors for *Salmonella* Enteritidis and Typhimurium (DT104 and non-DT104) infections in The Netherlands: Predominant roles for raw eggs in Enteritidis and sandboxes in Typhimurium infections. Epidemiol. Infect..

[157] Lucerón C.O., Meixeira A.P., Sanz I.A., Deleyto V.C., León S.H., Ruiz L.G. (2017). Notes from the field: An outbreak of *Salmonella* Typhimurium associated with playground sand in a preschool setting—Madrid, Spain, September-October 2016. MMWR Morb. Mortal. Wkly. Rep..

[158] Staff M., Musto J., Hogg G., Janssen M., Rose K. (2012). Salmonellosis outbreak traced to playground sand, Australia, 2007–2009. Emerg. Infect. Dis..

[159] Shibata T., Solo-Gabriele H.M. (2012). Quantitative microbial risk assessment of human illness from exposure to marine beach sand. Environ. Sci. Technol..

[160] Casadevall A. (2022). Immunity to invasive fungal diseases. Annu. Rev. Immunol..

[161] Nnadi N.E., Carter D.A. (2021). Climate change and the emergence of fungal pathogens. PLoS Pathog..

[162] Weiskerger C.J., Brandão J., Ahmed W., Aslan A., Avolio L., Badgley B.D., Boehm A.B., Edge T.A., Fleisher J.M., Heaney C.D. (2019). Impacts of a changing earth on microbial dynamics and human health risks in the continuum between beach water and sand. Water Res..

[163] Roca M.A., Brown R.S., Solo-Gabriele H.M. (2019). Fecal indicator bacteria levels at beaches in the Florida Keys after Hurricane Irma. Mar. Pollut. Bull..

